# Bridging Methodology and Clinical Relevance: Toward More Robust Evidence for Antioxidant Therapy in Male Infertility

**DOI:** 10.1590/S1677-5538.IBJU.2025.0407

**Published:** 2025-08-05

**Authors:** Shengyi Chen, Yuekun Fang, Bin Cheng

**Affiliations:** 1 Wenzhou Hospital of Integrated Traditional Chinese and Western Medicine Department of Andrology Wenzhou China Department of Andrology, Wenzhou Hospital of Integrated Traditional Chinese and Western Medicine, Wenzhou, China; 2 Wenzhou Hospital of Integrated Traditional Chinese and Western Medicine Department of Urology Wenzhou China Department of Urology, Wenzhou Hospital of Integrated Traditional Chinese and Western Medicine, Wenzhou, China

To the editor,

We read with great interest the recent article titled "Efficacy of Alpha Lipoic Acid Supplementation in Sperm Parameters: A Systematic Review and Meta-Analysis of Randomized Trials," published in the International Brazilian Journal of Urology ( 1 ). The authors should be commended for synthesizing data from five randomized controlled trials to assess the impact of alpha lipoic acid (ALA) on sperm quality in men with infertility. Their analysis, which demonstrated improvements in progressive motility, total motility, and abnormal morphology, provides valuable evidence supporting the growing interest in antioxidant therapy for male infertility. The study's rigorous adherence to PRISMA guidelines, use of GRADE criteria to assess evidence certainty, and implementation of sensitivity analyses further enhance its methodological credibility.

Despite the strengths of this meta-analysis, we would like to highlight several critical limitations not fully addressed by the authors and suggest directions for future research. First, while the study focuses on sperm parameters as surrogate outcomes, it does not clarify whether these improvements translate into meaningful reproductive endpoints. Enhanced motility or morphology does not necessarily correlate with successful fertilization, implantation, or live birth. Future systematic reviews should prioritize functional sperm outcomes, such as the DNA fragmentation index (DFI) ( 2 ), acrosome reaction capacity, and mitochondrial membrane potential. Additionally, where possible, clinical outcomes such as pregnancy and live birth rates should be included ( 3 ). These endpoints are more directly associated with reproductive success and would significantly enhance the clinical relevance of the findings.

Second, the included trials demonstrated considerable heterogeneity regarding baseline semen quality, potential infertility etiologies, and inclusion criteria (e.g., presence or absence of varicocelectomy). However, the meta-analysis did not perform subgroup analyses based on underlying causes of infertility. Since the antioxidant effects of ALA might vary according to oxidative stress levels, pooling all patients under the category of "idiopathic infertility" may obscure different treatment responses. Future studies should stratify patients by identifiable risk factors (e.g., metabolic syndrome, varicocele, smoking status) or baseline reactive oxygen species (ROS) levels to determine who would benefit most from ALA supplementation ( 4 ).

Third, the meta-analysis lacks consideration of inter-laboratory variability in semen analysis, a well-recognized issue in andrology ( 5 ). The reproducibility of parameters such as sperm concentration or morphology can be highly sensitive to sample handling, technician experience, and timing of analysis. Without standardized protocols or external quality controls across included trials, the reliability of pooled outcomes may be compromised. Future meta-analyses should preferentially include trials adhering to WHO laboratory guidelines or those employing validated semen analysis protocols.

In summary, the authors have significantly contributed to the literature on antioxidant therapy in male infertility, and their findings support the potential role of ALA in improving specific sperm parameters. However, to better inform clinical practice, future research should emphasize functionally relevant outcomes, account for etiological heterogeneity, and ensure methodological standardization across studies. These enhancements will offer clearer insights into whether ALA supplementation should be routinely recommended for men with idiopathic infertility.

## Data Availability

Not applicable
